# Comparative Motor Outcomes and Programming Timelines After Deep Brain Stimulation for Parkinson’s Disease With Abbott Infinity™ Versus Medtronic Percept™ Systems

**DOI:** 10.7759/cureus.115177

**Published:** 2026-08-25

**Authors:** Patrick Pema, Triston Messer, Jake Barsch, Jake Heath, Yen-Hong Kuo, Burhanuddin Danish, Dana Dolce, Nitesh V Patel, Shabbar Danish, Jasdeep S Hundal

**Affiliations:** 1 Neurosurgery, Hackensack Meridian Health, Hackensack, USA; 2 Neurosurgery, Hackensack Meridian School of Medicine, Nutley, USA; 3 Epidemiology and Public Health, Hackensack Meridian Health Jersey Shore University Medical Center, Neptune Township, USA; 4 Neurosurgery, Rutgers University, Newark, USA; 5 Neuropsychiatry, Hackensack Meridian Health Jersey Shore University Medical Center, Neptune Township, USA; 6 Neurosurgery, Hackensack Meridian Health Jersey Shore University Medical Center, Neptune Township, USA; 7 Neurology, Hackensack Meridian Health Jersey Shore University Medical Center, Neptune Township, USA

**Keywords:** abbott infinity, deep brain stimulation, mds-updrs, medtronic percept, parkinson's disease

## Abstract

Introduction

Deep brain stimulation (DBS) is an established therapy for the management of advanced Parkinson’s disease, contributing to improvements in motor symptoms and quality of life. Medtronic Percept™ (Medtronic plc, Galway, Ireland) and Abbott Infinity™ (Abbott Laboratories, Abbott Park, Illinois, United States) DBS systems are both widely used by clinicians yet lack real-world head-to-head comparisons of postoperative outcomes. This study evaluated motor outcomes, dopamine challenge responsiveness, cognitive baseline measures, and time to initial programming of patients implanted with Medtronic versus Abbott DBS systems.

Methods

We performed an exploratory retrospective cohort study of patients with Parkinson’s disease who underwent primary DBS implantation with either the Abbott Infinity or Medtronic Percept systems between October 2021 and November 2024 at Jersey Shore University Medical Center, New Jersey, United States. Thirty-nine patients were screened, of whom six were excluded for not meeting inclusion criteria, leaving 33 patients for analysis. Between-group comparisons were performed using nonparametric tests. Longitudinal postoperative Unified Parkinson’s Disease Rating Scale Part III (UPDRS) motor scores were evaluated using a generalized estimating equation model accounting for repeated measurements within patients and adjusting for baseline UPDRS Part III score. Exploratory within-group comparisons were performed using Wilcoxon signed-rank tests with Holm correction for multiple comparisons.

Results

A total of 33 patients met inclusion criteria; these included 10 Abbott Infinity and 23 Medtronic Percept recipients. Baseline characteristics were similar between groups, including age, sex, and preoperative UPDRS motor scores. Postoperative UPDRS motor scores did not differ significantly between patients with Abbott Infinity or Medtronic Percept at three months, six months, nine months, or 12 months. Longitudinal analysis demonstrated no significant device-by-time interaction (p = 0.608). At 12 months, median UPDRS motor score was 10.5 in the Abbott group and 8.0 in the Medtronic group (p = 0.969). Dopamine challenge OFF scores, ON scores, percent improvement, baseline Dementia Rating Scale scores, and time to initial programming were also not significantly different between groups. Median time to initial programming was 27.5 days for Abbott Infinity and 25.0 days for Medtronic Percept (p = 0.317).

Conclusion

In this single-center exploratory retrospective cohort, no statistically significant differences were detected between Abbott Infinity and Medtronic Percept DBS systems in postoperative motor outcome trajectories, dopamine challenge profiles, baseline cognitive measures, or time to initial programming. Given the small and imbalanced cohort, these findings should not be interpreted as demonstrating equivalence between devices. Larger, longer-term prospective multi-center studies are needed to determine whether clinically meaningful device-specific differences exist.

## Introduction

Deep brain stimulation (DBS) is a recommended and proven treatment option for patients with advanced Parkinson’s disease unresponsive to medical therapy and who have been deemed medically fit for surgery [1-4]. PD is characterized by the degeneration of dopaminergic neurons in the substantia nigra, causing a disruption in the electrical circuitry between the basal ganglia, thalamus, and cortex. The loss of dopamine from the substantia nigra leads to increased activity of the indirect pathway of the basal ganglia, which is normally responsible for inhibiting movement. This increase in indirect pathway activity is due to decreased striatal inhibition of the globus pallidus externa, leading to the pathologic amplification of erroneous signalling. Current literature highlights how DBS implanted in the subthalamic nucleus improves motor function and quality of life for Parkinson’s patients [1,5,6]. DBS therapy in this manner has been shown to improve tremor, rigidity, and bradykinesia as well as decreasing “off” periods. The result is an increase in the patient’s mobility, autonomy, and quality of life [6-8]. Additional benefits include a reduction in both dosage of dopaminergic medications and their associated side effects [6].

An implantable DBS unit targets specific structures within the basal ganglia with the goal of improving the aforementioned motor pathway function. Although its mechanism remains incompletely understood, DBS is thought to modulate pathological activity and abnormal oscillations within basal ganglia-thalamocortical networks [9,10]. Thus, each implanted unit requires modulation of electrical stimuli tailored to the specific patient.

The Medtronic Percept™ family of DBS devices (Medtronic plc, Galway, Ireland) incorporates BrainSense™ technology, which allows the system to record local field potential activity that may provide additional information to guide clinician-directed programming [11]. Medtronic DBS technology has been studied extensively, with literature demonstrating favorable motor and quality of life outcomes following DBS treatment [12-14].

Abbott Infinity™ DBS system (Abbott Laboratories, Abbott Park, Illinois, United States) similarly provides clinician-directed stimulation and incorporates segmented directional lead technology, allowing current steering and adjustment of the stimulation field. Implantation of the Infinity system has also been associated with favorable motor outcomes in patients undergoing DBS [15].

Current literature contains limited real-world, head-to-head comparative data between commonly used DBS systems in routine clinical practice. Given the positive clinical outcomes associated with both devices, a comparative analysis may help identify clinically meaningful differences and inform patient-centered device selection. Device-specific factors may also affect patient experience and postoperative management. This study retrospectively evaluated patient outcomes and characterize motor symptom management associated with different DBS devices at Jersey Shore University Medical Center (JSUMC), and provided exploratory comparative data to support future research on precision in neuromodulation treatment strategies, and. We hypothesized that device type could be associated with differences in postoperative motor outcomes or time to initial programming.

## Materials and methods

This was an exploratory retrospective cohort study of patients with Parkinson’s disease who underwent primary DBS implantation with either the Medtronic Percept or Abbott Infinity DBS systems between October 2021 and November 2024 at Jersey Shore University Medical Center, Neptune Township, New Jersey, United States. The study was approved by the Hackensack Meridian Health Institutional Review Board (approval number: Pro 2024-0345). Informed consent was waived by the Institutional Review Board for this retrospective study.

Study population

In total, 39 patients were screened for inclusion. Inclusion criteria were: (i) a documented diagnosis of primary Parkinson’s disease, (ii) primary DBS implantation for Parkinson’s disease between October 2021 and November 2024, (iii) available preimplantation original Unified Parkinson’s Disease Rating Scale (UPDRS) Part III motor score and postoperative scores at approximately three, six, nine, and 12 months, and (iv) an available baseline Dementia Rating Scale-2 (DRS-2) assessment. Patients were required to meet all four inclusion criteria. Exclusion criteria included prior DBS implantation or revision, atypical or secondary parkinsonism, and incomplete baseline or follow-up data. Six patients were excluded for not meeting the inclusion criteria, leaving 33 unique patients in the final analysis.

DBS device allocation was not randomized and was based on surgeon preference.

Data collection

Demographic and clinical data were obtained by retrospective review of the electronic medical record. Motor function was assessed using Part III (Motor Examination) of the original UPDRS [16]. Variables included age at implantation, sex, DBS device type, baseline UPDRS Part III motor score, and postoperative UPDRS Part III motor scores at approximately three, six, nine, and 12 months. An acceptable assessment window of within one week of each scheduled follow-up time point was used. Postoperative motor assessments were performed in the stimulation ON state during routine clinical follow-up visits. Medication state and timing relative to dopaminergic dosing were not standardized during postoperative assessments.

Dopamine challenge data included OFF-medication and ON-medication motor scores and percent improvement. Percent improvement was calculated as: $\left[\frac{\text{OFF score} - \text{ON score}}{\text{OFF score}}\right] \times 100$.

Baseline cognitive function was assessed using the DRS-2, including the raw total score and age-corrected Mayo’s Older Americans Normative Studies (MOANS) scaled scores [17]. Time to initial programming was defined as the interval, in days, from implantation to the first programming session.

Outcomes

The primary outcome was postoperative motor function as measured by original UPDRS Part III motor scores at serial follow-up intervals. Secondary outcomes included motor responsiveness during dopamine challenge testing and time to initial programming. Exploratory outcomes included within-group change from baseline and the proportion of patients achieving at least 20% and 30% reductions in UPDRS Part III score.

Statistical analysis

Statistical analyses were performed using IBM SPSS Statistics for Windows, version 31 (IBM Corp., Armonk, New York, United States). Continuous variables were assessed for normality using the Shapiro-Wilk test. As most variables were non-normally distributed, continuous data are presented as medians with interquartile ranges (IQRs), and nonparametric tests were used for comparisons.

Between-group differences (Abbott vs Medtronic) were evaluated using the Mann-Whitney U test for continuous variables and Fisher’s exact test for categorical variables, as appropriate. Comparisons included baseline characteristics, postoperative original UPDRS Part III scores at each follow-up interval, dopamine challenge outcomes, baseline DRS scores, and time to initial programming.

To account for repeated motor assessments within patients, postoperative UPDRS Part III scores were additionally evaluated using a generalized estimating equation model with patient as the repeated subject and an exchangeable working correlation structure. Baseline UPDRS Part III score, device type, postoperative time point, and the device-by-time interaction were included in the model. Time was modeled categorically, and adjusted between-device differences are reported with 95% confidence intervals (CIs).

Exploratory within-group comparisons between baseline and postoperative UPDRS Part III scores were performed using Wilcoxon signed-rank tests. Holm correction was applied across the four postoperative time points within each device group. Percentage change from baseline was calculated as: $\left[\frac{\text{Baseline score} - \text{Postoperative score}}{\text{Baseline score}}\right] \times 100$.

One Abbott patient with a baseline UPDRS Part III score of 0 was excluded from percentage-change and responder analyses because percentage change could not be calculated. Responder analyses evaluated the proportion of patients achieving at least 20% and 30% reductions from baseline and were compared using Fisher’s exact test. All tests were two-sided, and statistical significance was defined as p < 0.05.

## Results

A total of 33 patients were included in this study; 10 received Abbott Infinity, and 23 received Medtronic Percept implants. Median age was 60.5 years in the Abbott group and 67.0 years in the Medtronic group. There were five female patients (50%) in the Abbott cohort and 10 (43.5%) in the Medtronic cohort. Baseline demographic characteristics for age (p = 0.095) and sex (p = 1.000) were similar between groups. Preoperative baseline motor scores did not differ significantly between groups (p = 0.695). These results are described in Table 1.

**Table 1:** Baseline characteristics, UPDRS motor and dopamine challenge scores, dementia rating scale values, and time to initial programming for the two groups Data are presented as median (IQR), except for sex, which is presented as n (%). Between-group comparisons used the Mann-Whitney U test for continuous variables and Fisher’s exact test for sex. UPDRS: Unified Parkinson’s Disease Rating Scale; IQR: interquartile range; MOANS: Mayo’s Older Americans Normative Studies

Variable		Abbott Group (n=10), median (IQR)	Medtronic Group (n=23), median (IQR)	p-value
Age (years)		60.5 (55.0, 69.3)	67.0 (63.5, 71.0)	0.095
Sex, n (%)	Female	5 (50.0%)	10 (43.5%)	1.000
	Male	5 (50.0%)	13 (56.5%)	
Baseline UPDRS Part III Motor Score		11.3 (3.63, 37.0)	10.0 (5.00, 17.0)	0.695
UPDRS Part III, 3 months		11.5 (2.75, 28.1)	7.00 (6.00, 13.0)	0.709
UPDRS Part III, 6 months		14.5 (2.50, 31.0)	8.00 (4.75, 13.3)	0.367
UPDRS Part III, 9 months		13.0 (2.13, 18.0)	10.0 (5.00, 15.3)	0.906
UPDRS Part III, 12 months		10.5 (1.13, 17.1)	8.00 (4.00, 13.0)	0.969
Dopamine challenge OFF score		36.5 (29.9, 55.5)	36.4 (30.8, 46.3)	0.754
Dopamine challenge ON score		14.3 (11.0, 22.0)	11.5 (8.00, 14.5)	0.357
Dopamine challenge improvement, %		65.9 (54.4, 73.5)	70.0 (60.8, 76.2)	0.518
Time to initial programming, days		27.5 (24.0, 35.3)	25.0 (20.0, 36.5)	0.317
Baseline Dementia Rating Scale Raw Score		137.5 (136, 139)	136 (132.5, 139)	0.270
Baseline Dementia Rating Scale age-corrected MOANS scaled score		9.50 (9.00, 10.0)	9.00 (8.00, 10.0)	0.378

**Figure 1:**
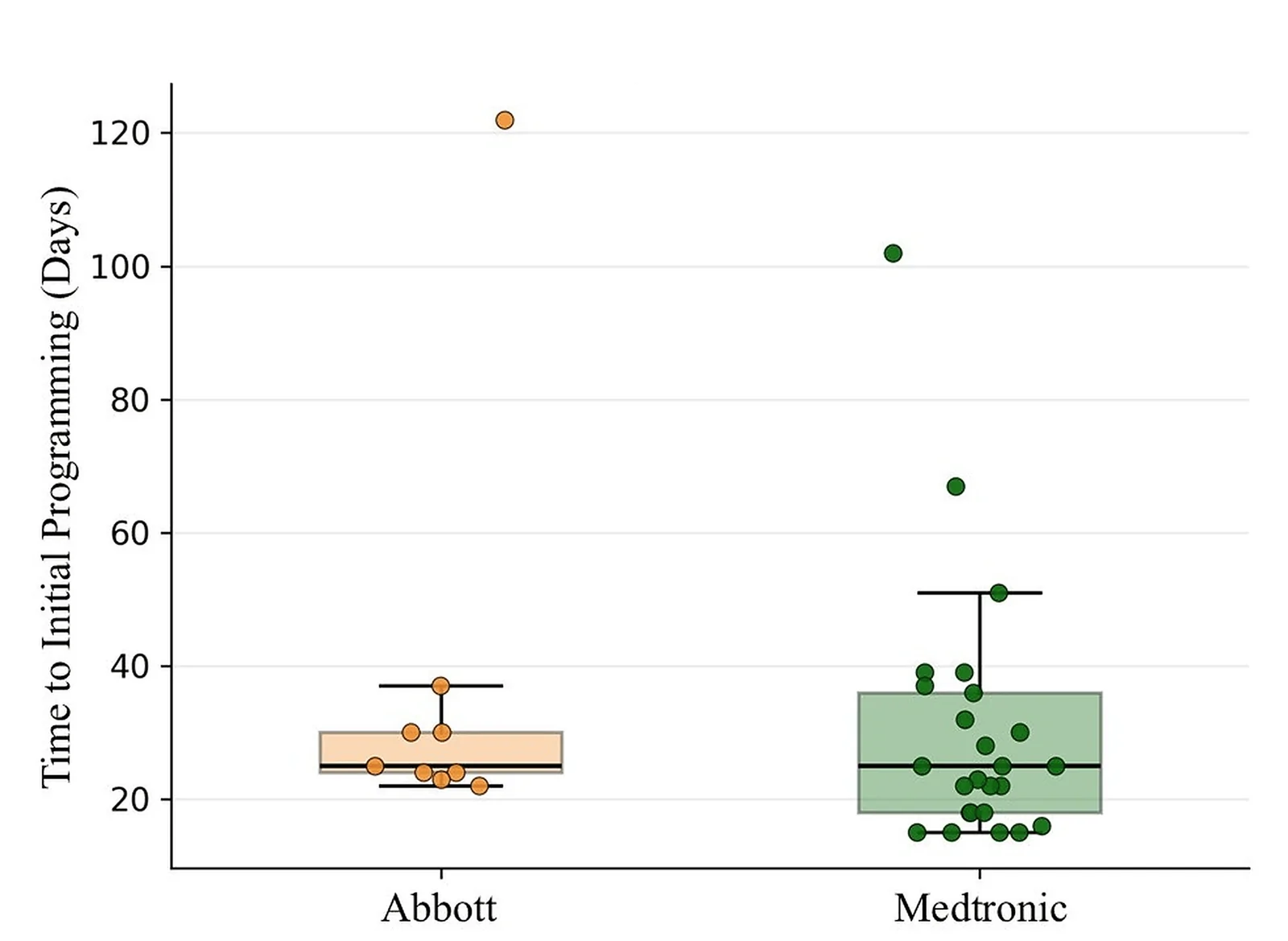
Box plot with jittered data points of time to initial programming for Abbott Infinity* and Medtronic Percept** devices Boxes represent the interquartile range, center lines represent medians, whiskers represent values within 1.5 times the interquartile range, and points represent individual patients. The between-group comparison used the Mann-Whitney U test (p = 0.317; also reported in Table 1). *Abbott Laboratories, Abbott Park, Illinois, United States; **Medtronic plc, Galway, Ireland

UPDRS motor outcome trends are depicted in Figure 1.. Part III motor scores did not differ significantly between the two sets of patients at three, six, nine, or 12 months (Table 2). The median 12-month motor score was 10.5 in the Abbott group and 8.0 in the Medtronic group (p = 0.969). Median percent improvement during dopamine challenge testing was 65.9% for Abbott patients and 70.0% for Medtronic patients (p = 0.518). Median time to initial programming was 27.5 days for the Abbott group and 25.0 days for the Medtronic group (p = 0.317). Dopamine challenge OFF-medication scores (p = 0.754), ON-medication scores (p = 0.357), baseline DRS-2 raw scores (p = 0.270), and DRS-2 age-corrected MOANS scaled scores (p = 0.378) also did not differ significantly between groups.

**Table 2:** Exploratory UPDRS Part III percentage change and responder analysis Percentage change was calculated as $\frac{\text{Baseline UPDRS Part III} - \text{Postoperative UPDRS Part III}} {\text{Baseline UPDRS Part III}} \times 100$. One Abbott patient with a baseline UPDRS Part III score of 0 was excluded from percentage-change and responder analyses. Between-group percentage-change comparisons used the Mann-Whitney U test, and responder proportions were compared using Fisher’s exact test. UPDRS: Unified Parkinson’s Disease Rating Scale

Follow-up	Abbott Group, median % change (IQR)	Medtronic Group, median % change (IQR)	p-value	≥20% Reduction Abbott Group (n=9)	≥20% Reduction Medtronic Group (n=23)	p-value	≥30% Reduction Abbott Group (n=9)	≥30% Reduction Medtronic Group (n=23)	p-value
3 months	24.4 (−18.2, 35.2)	0.0 (−20.0, 42.3)	0.883	5/9 (55.6%)	9/23 (39.1%)	0.453	3/9 (33.3%)	8/23 (34.8%)	1.000
6 months	19.5 (0.0, 33.3)	0.0 (−40.0, 27.5)	0.240	4/9 (44.4%)	9/23 (39.1%)	1.000	3/9 (33.3%)	6/23 (26.1%)	0.685
9 months	32.0 (−9.1, 78.0)	0.0 (−52.3, 18.6)	0.165	5/9 (55.6%)	6/23 (26.1%)	0.213	5/9 (55.6%)	5/23 (21.7%)	0.096
12 months	33.3 (6.8, 62.5)	0.0 (−10.0, 33.3)	0.130	6/9 (66.7%)	10/23 (43.5%)	0.433	6/9 (66.7%)	8/23 (34.8%)	0.132

Longitudinal analysis of postoperative UPDRS Part III scores demonstrated no statistically significant device-by-time interaction across three-, six-, nine-, and 12-month follow-ups (Wald χ² [3] = 1.83, p = 0.608). The adjusted Medtronic − Abbott difference was −0.85 points at three months (95% CI −4.44 to 2.75), −1.99 points at six months (95% CI −6.68 to 2.70), −1.19 points at nine months (95% CI −8.38 to 6.01), and 0.97 points at 12 months (95% CI −3.09 to 5.03). The confidence intervals included zero at each follow-up interval.

Exploratory within-group analyses did not demonstrate statistically significant changes in UPDRS Part III scores from baseline at any postoperative time point after Holm correction. At 12 months, the median change from baseline was −2.75 points in the Abbott group (Holm-adjusted p = 0.281) and 0.0 points in the Medtronic group (Holm-adjusted p = 0.368).

## Discussion

UPDRS scoring is a significant outcome measure in Parkinson’s disease, helping providers to assess functional status, guide patient selection, and determine treatment efficacy [16,18]. This measure also allows comparisons in motor symptom severity between different patient populations across different studies and centers. Literature demonstrates that patients may experience motor symptom improvement at three- and six-month follow-up assessments; our study revealed no statistically detectable differences in UPDRS scores between devices at those time periods. Within-group analyses did not demonstrate statistically significant changes from baseline after correction for multiple comparisons. Moreover, no significant between-device differences were detected at any time period. These findings should not be interpreted as demonstrating equivalent motor outcomes between devices.

**Figure 2:**
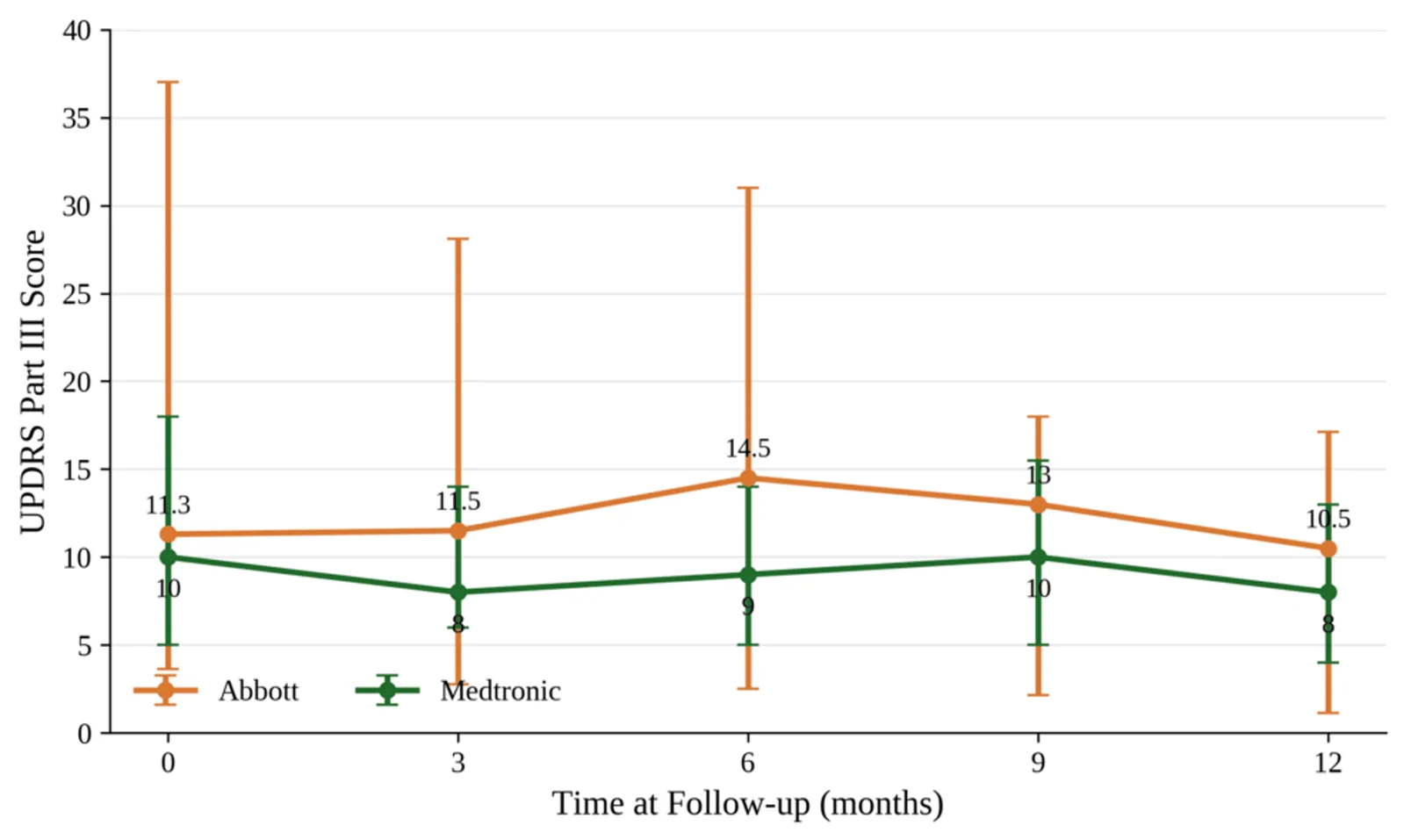
Median UPDRS Part III motor scores by DBS device type at baseline and 3-, 6-, 9-, and 12-month follow-ups Points represent medians and error bars represent interquartile ranges. Between-group comparisons used the Mann-Whitney U test; p = 0.695, 0.709, 0.367, 0.906, and 0.969 at baseline and three, six, nine, and 12 months, respectively (also reported in Table 1). Longitudinal analysis using a generalized estimating equation model demonstrated no significant device-by-time interaction (p = 0.608). UPDRS: Unified Parkinson’s Disease Rating Scale; DBS: deep brain stimulation

Motor score trajectories were also broadly similar descriptively between the two groups. As shown in Figure 2, median scores fluctuated modestly over time, and no statistically significant between-group differences were detected at baseline or at any postoperative follow-up. Longitudinal analysis similarly demonstrated no statistically significant device-by-time interaction. The Abbott group median scores increased slightly through six months and then decreased by 12 months, whereas the Medtronic group median scores decreased at three months, increased modestly through nine months, and decreased again at 12 months. Given the small cohort, wide IQRs, and wide CIs around between-device estimates, these descriptive patterns should not be interpreted as evidence of device-specific temporal effects.

Time to initial programming influences postoperative DBS management for every patient. While optimization of stimulation typically occurs in the months following implantation, initial programming is commonly performed during the early postoperative period [19]. For Parkinson’s disease, this step is essential for a patient’s quality of life and has a direct impact on how soon appropriate modifications may occur. As such, shorter time to programming may facilitate quicker alleviation of symptoms and earlier refinement of device parameters at subsequent visits. In our study, Medtronic and Abbott devices demonstrated no significant difference in time to initial programming. Time to initial programming is also likely influenced by device-independent factors, including clinician expertise, programming strategy, postoperative recovery, appointment availability, and center-specific workflow [19-21]. Therefore, this outcome should not be interpreted as an intrinsic measure of device performance.

The acute levodopa challenge quantifies motor responsiveness to dopaminergic therapy and is commonly incorporated into the evaluation of DBS candidates [22,23]. In this study, similar OFF-medication scores, ON-medication scores, and percent improvement were observed between the Abbott and Medtronic cohorts, suggesting that measured baseline levodopa responsiveness was not significantly different between groups [22,23]. Similarly, no significant difference was observed in DRS-2 values. Comparable dopamine challenge and DRS-2 results reduce the likelihood that major measured differences in baseline dopaminergic responsiveness or cognitive status explain the postoperative findings. However, other potentially relevant baseline disease characteristics were not available for analysis.

Because the factors determining device selection were not systematically measured beyond surgeon preference, the influence of other factors on device allocation cannot be determined. The study was not designed to establish functional equivalence between devices.

Limitations

Several limitations should be acknowledged. Follow-up duration in this study was limited to 12 months postoperatively; future studies should explore motor status trends at longer-term follow-ups. Additionally, our sample size was 33 patients, with an imbalance between the Abbott and Medtronic cohorts of 10 versus 23 patients, respectively. This limited statistical power and prevents conclusions regarding equivalence or noninferiority between devices. Larger cohort studies may be able to elucidate clinically meaningful differences in our measured variables.

This single-center retrospective study reflects outcomes of patients with Parkinson’s disease at Jersey Shore University Medical Center. Device selection was not randomized and was based on surgeon preference, introducing the possibility of selection bias and unmeasured confounding by indication. Requiring longitudinal follow-up may also have preferentially selected patients who maintained consistent postoperative care. Multi-center studies could shed light on different programming strategies and provider-specific outcomes.

Several potentially important clinical variables were not available in the analytic dataset, including surgical target, laterality, whether implantation was staged or performed as a single-stage procedure, longitudinal stimulation parameters, changes in levodopa equivalent daily dose, use of device-specific algorithms, disease duration, Hoehn-Yahr stage, and predominant symptom type or side. Medication state and timing relative to dopaminergic dosing during routine postoperative UPDRS assessments were not standardized, which may have influenced measured motor scores. Information regarding assessor blinding was also unavailable.

Safety and complication outcomes were not evaluated in this study. Finally, time to initial programming is substantially influenced by institutional workflow, appointment availability, and clinical practice patterns and therefore should not be considered an intrinsic characteristic of the DBS device. Larger prospective, multi-center studies with standardized assessment and programming protocols are needed to determine whether clinically meaningful device-specific differences exist.

## Conclusions

As the DBS field continues to grow and broaden to different pathologies, expanding the body of literature surrounding each device’s mechanism of action and postoperative outcomes is of the utmost importance. Identifying relevant patient-, provider-, and center-specific characteristics that may ultimately influence management is essential to understanding how best to approach operative intervention. In the current study, no statistically significant differences were detected between Abbott and Medtronic devices in postoperative UPDRS Part III trajectories, dopamine challenge measures, baseline cognitive measures, or time to initial programming. These findings should not be interpreted as demonstrating equivalent outcomes between devices. Larger, prospective, and ideally multi-center studies are required to determine whether clinically meaningful device-specific differences exist. This study provides exploratory data regarding device selection and postoperative motor trajectories and contributes to our understanding of Parkinson’s disease management.
